# Biomechanical comparison of unilateral and bilateral pedicle screws fixation for transforaminal lumbar interbody fusion after decompressive surgery -- a finite element analysis

**DOI:** 10.1186/1471-2474-13-72

**Published:** 2012-05-16

**Authors:** Shih-Hao Chen, Shang-Chih Lin, Wen-Chi Tsai, Chih-Wei Wang, Shih-Heng Chao

**Affiliations:** 1 Department of Orthopaedics, Tzu-Chi General Hospital at Taichung and Tzu Chi University, Hualien, Taiwan; 2 Graduate Institute of Biomedical Engineering, National Taiwan University of Science and Technology, Taipei, Taiwan; 3 BoneCare Orthopedic Centers, Han-Chiung Clinics, Taipei, Taiwan; 4 Department of Mechanical Engineering, National Chiao-Tung University, Hsinchu, Taiwan

**Keywords:** Transforaminal lumbar interbody fusion, Pedicle screw fixation, Contralateral facet screw, Finite element analysis

## Abstract

**Background:**

Little is known about the biomechanical effectiveness of transforaminal lumbar interbody fusion (TLIF) cages in different positioning and various posterior implants used after decompressive surgery. The use of the various implants will induce the kinematic and mechanical changes in range of motion (ROM) and stresses at the surgical and adjacent segments. Unilateral pedicle screw with or without supplementary facet screw fixation in the minimally invasive TLIF procedure has not been ascertained to provide adequate stability without the need to expose on the contralateral side. This study used finite element (FE) models to investigate biomechanical differences in ROM and stress on the neighboring structures after TLIF cages insertion in conjunction with posterior fixation.

**Methods:**

A validated finite-element (FE) model of L1-S1 was established to implant three types of cages (TLIF with a single moon-shaped cage in the anterior or middle portion of vertebral bodies, and TLIF with a left diagonally placed ogival-shaped cage) from the left L4-5 level after unilateral decompressive surgery. Further, the effects of unilateral versus bilateral pedicle screw fixation (UPSF *vs.* BPSF) in each TLIF cage model was compared to analyze parameters, including stresses and ROM on the neighboring annulus, cage-vertebral interface and pedicle screws.

**Results:**

All the TLIF cages positioned with BPSF showed similar ROM (<5%) at surgical and adjacent levels, except TLIF with an anterior cage in flexion (61% lower) and TLIF with a left diagonal cage in left lateral bending (33% lower) at surgical level. On the other hand, the TLIF cage models with left UPSF showed varying changes of ROM and annulus stress in extension, right lateral bending and right axial rotation at surgical level. In particular, the TLIF model with a diagonal cage, UPSF, and contralateral facet screw fixation stabilize segmental motion of the surgical level mostly in extension and contralaterally axial rotation. Prominent stress shielded to the contralateral annulus, cage-vertebral interface, and pedicle screw at surgical level. A supplementary facet screw fixation shared stresses around the neighboring tissues and revealed similar ROM and stress patterns to those models with BPSF.

**Conclusions:**

TLIF surgery is not favored for asymmetrical positioning of a diagonal cage and UPSF used in contralateral axial rotation or lateral bending. Supplementation of a contralateral facet screw is recommended for the TLIF construct.

## Background

The goal of lumbar interbody fusion surgery is to achieve a solid arthrodesis of spinal segments that can sustain loading, while maintaining proper disc space height, preserving foraminal dimensions, and restoring sagittal plane alignment [1-4]. Currently, interbody fusion techniques have been refined with regard to surgical approach, instrumentation, and bone graft material [5-7]. Posterior lumbar interbody fusion (PLIF) has been scrutinized in the field of decompressive surgery because of incidence of iatrogenic dural tear, epidural bleeding, neural injury, risk of damage to cauda equina at higher levels as well as adjacent soft tissue severance, including muscles, facet joints, and ligamentous structures [1,2,8]. In 1982, Harms and Rolinger introduced TLIF as an alternate technique, which involved removal of one facet joint and a more lateral approach to the disc space, thus reducing the potential for nerve injury, particularly in the presence of scarring after prior surgery [9]. Bilateral pedicle screw fixation (BPSF) is used by rod contouring to restore lumbar lordosis and disc height, while interbody fusion cage is positioned under compression in the anterior or middle column. The principles of TLIF technique require less bone and soft tissue dissection, respect neural elements, spare the spinal process ligaments, lamina, facet joint and pars interarticularis on the contralateral side for additional posterolateral fusion, and avoid the morbidity of anterior-posterior approaches [10-13]. Clinical studies indicate that TLIF is equivalent or even superior to PLIF regarding outcome, fusion rate and complications [11-13].

Recently, unilateral pedicle screw fixation (UPSF) has been used in the minimally invasive TLIF procedure to provide construct stability for fusion, minimize access for decompression, and reduce approach-related morbidity related to muscle injury and implant load [11,14,15]. This novel procedure using paramedian incision unilaterally has several advantages, including no tissues dissection on the contralateral side, less postoperative pain, and quicker patient recovery [14-17]. Chen *et al*. used an *in vitro* porcine model to study the biomechanical behavior of interbody cages with unilateral or bilateral pedicle screw instrumentation, and revealed that UPSF with two-cage implantation was a stronger construct than the intact model to maintain stability for fusion [18]. However, a degree of motion and stress redistribution exists following decompression and TLIF with unilateral instrumentation, which might influence spinal stiffness for fusion and degeneration over time [19-22]. The combination of a contralateral translaminar facet screw with UPSF is under increased investigation [13]. A validated finite element (FE) model has the advantages of easily modifying TLIF cage and posterior implant geometry to observe the altered load transfer on the individual motion segment, and to analyze the stress distribution on the neighboring structures [21,23-27]. Few studies have investigated the biomechanical performance of TLIF procedures when different cage positioning and posterior implants are used, as compared with the traditional methods. Therefore, the aim of this study was to identify the kinematic and mechanical effects of various instrumentations through a single point access for TLIF based on a simulation analysis. The current authors compared the ROM and stress of different TLIF cage models provided by UPSF versus BPSF on the effectiveness of spinal kinematics relative to the normative values. The necessity of supplementary facet screw fixation was hypothesized and evaluated for the TLIF construct.

## Methods

**Figure 1:**
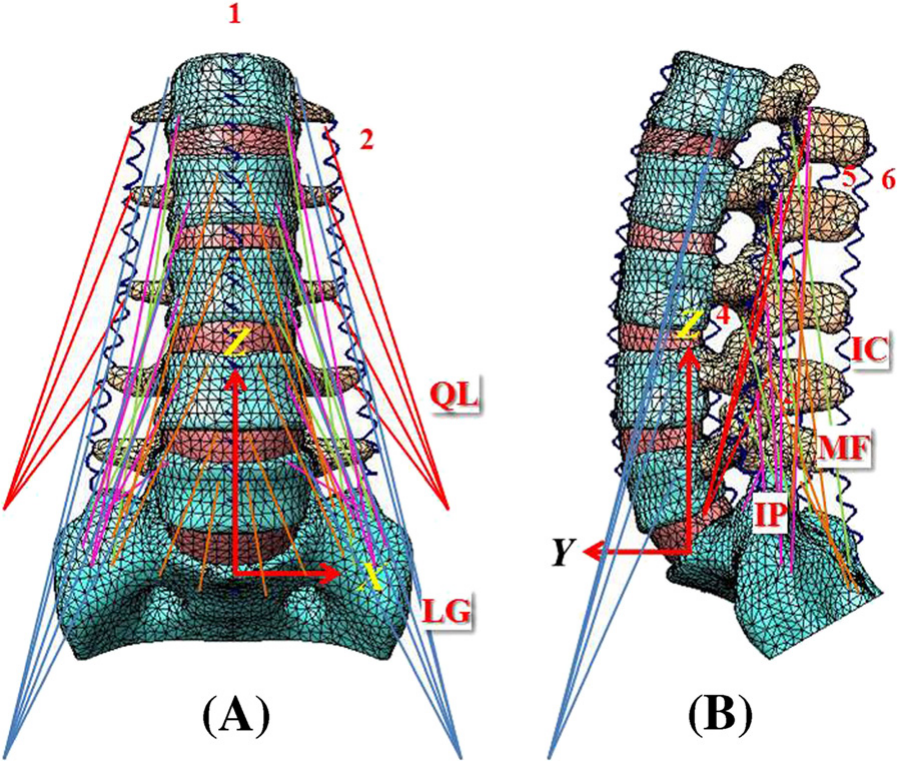
** The finite-element model used in this study. (A)** Front view. **(B)** Lateral view.

**Figure 2:**
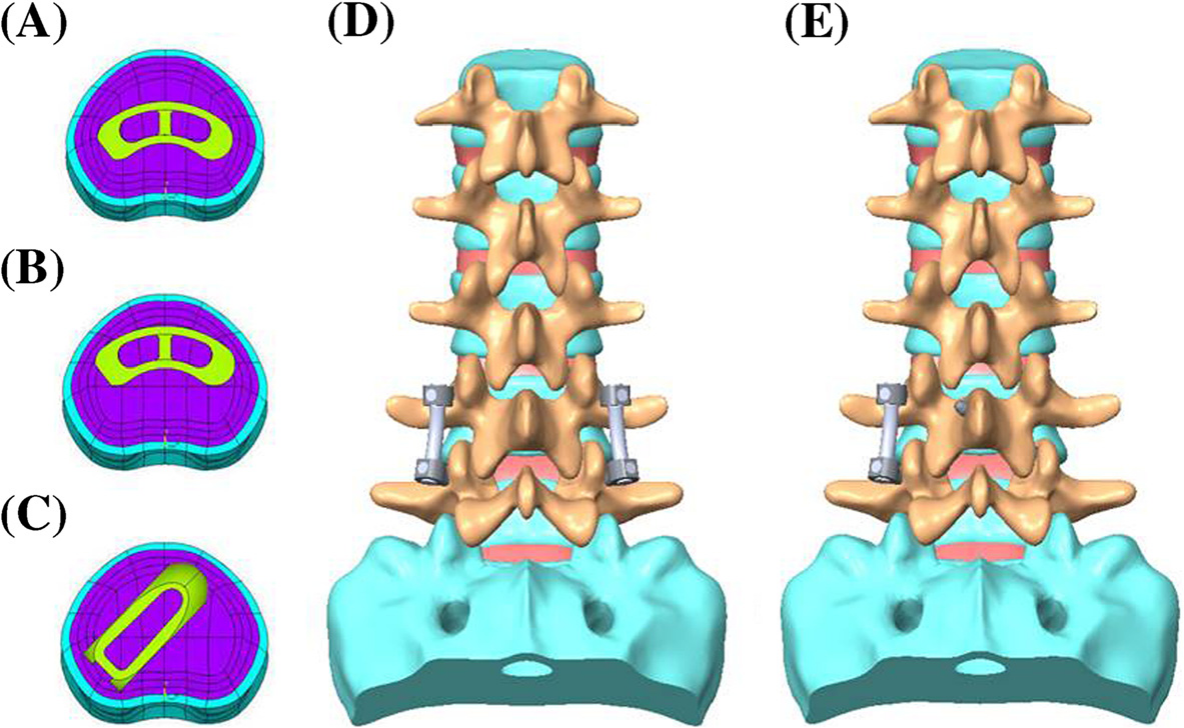
Finite element models show: Intact model of the L1-S1 segments; transforaminal lumbar interbody fusion (TLIF) cage implanted in models: (A) TLIFm (moon-shaped cage; middle); (B) TLIFa (moon-shaped cage; anterior); (C) TLIFo (left diagonal cage); and supplemented with (D) Bilateral pedicle screw fixation after minimal invasive TLIF surgery; or (E) Unilateral pedicle screw plus translaminar facet screw fixation contralaterally in TLIFo model (TLIFo + f).

This study involved the establishment of a FE model of intact L1 to S1 (INT model) (Figure 1). Its modification allows for implantation of the following types of interbody cages at the left L4-5 level after unilateral decompressive surgery (Figure 2). The variations of implantation were given: TLIF with a ogival-shaped cage (11 × 12 × 30 mm × 4°; polyetheretherketon (PEEK); O.I.C. cage, Stryker Orthopaedics, Mahwah, NJ) diagonally positioned at 45° (TLIFo), or TLIF with a single moon-shaped cage (11 × 12 × 30 mm × 4°; PEEK; AVS-TL cage, Stryker Orthopaedics) in the anterior or middle portion of vertebral bodies (TLIFa or TLIFm, respectively) (Figures 2C-E). Further, this study compared the effect of UPSF or BPSF on each of the TLIF cage models relative to the INT model (Figure 2A). A translaminar facet screw was contralaterally supplemented with UPSF in the TLIFo model (TLIFo + f) to evaluate its additional effect (Figure 2B).

### FE model of the intact lumbar spine

To create this model, the current authors obtained computed tomography scans of the L1-S1 lumbar spine at 1-mm intervals in a healthy, middle-aged man. “Written informed consent was obtained from the patient for publication of this report and any accompanying images”. The commercially available FE software, Simulation Ed. 2011 (SolidWorks Corporation, Concord, MA, USA) was used to evaluate differences between the intact and surgery models. The FE model of the osseoligamentous lumbar spine included vertebrae, intervertebral discs, endplates, posterior bony elements, and the following ligaments: anterior longitudinal ligament, posterior longitudinal ligament, interspinous ligament, intertransverse ligament, ligamentum flavum, and facet capsular ligament (Figure 1). The material properties of lumbar spine were assumed to be homogeneous and linear [26,28]. Ligaments were simulated as the tension-only spring, and elements were arranged in the anatomic direction. The 10-node solid elements were used for modeling the cortical bone, cancellous bone, endplate, posterior bony element and disc. The disc annulus consisted of fibers embedded in the ground substance. Facet joints were treated as a nonlinear three-dimensional contact program using surface-to-surface contact elements, and the friction coefficient was set at 0.1 [28].

### FE models of TLIF with instrumentation after decompressive surgery

To simulate the standard TLIF model, unilateral total facetectomy and partial discetomy were performed at the L4-5 level. The left facet joint, ligamentum flavum, posterolateral annulus, and total nucleus pulposus were removed, but the posterior bony elements, contralateral facet joint, and supraspinous and interspinous ligaments were preserved. The INT model was modified to this decompressive status, and then instrumented with TLIFa, TLIFm, or TLIFo cage between the L4 and L5 vertebrae, each covering a contact area of 254 to 260 mm^2^ (Table 1). Two or four simulated pedicle screws (diameter, 6 mm) were inserted through the pedicles of L4 and L5 vertebrae unilaterally or bilaterally, and connected by one or two rods (diameter: 6 mm) modeled with solid elements. These screw-bone interfaces were designed to be a full constraint. The cage-vertebra interface was modeled by surface-to-surface contact elements to simulate the early postoperative stage after spinal instrumentation. These contact elements transmitted compression force but not tension. The coefficient of friction at the cage-vertebra interface was 0.8 to mimic small teeth on the contact surfaces [21]. In addition, the contralateral facet screw (diameter, 3.5 mm) was designed to be fully constrained in the TLIFo + f model.

**Table 1:** Materials properties of implants used in the current FE models*

**Materials**	**Young’s modulus (Mpa)**	**Poisson’ ratio**
Stryker O.I.C. cage	3600	0.3
(PEEK)		
Stryker Moon-shaped cage	3600	0.3
(PEEK)		
Pedicle screws	110000	0.3
(Titanium alloy)		
Translaminar facet screw	110000	0.3
(Titanium alloy)		

*Material properties of the lumbar spine had been presented in previous students [26,33].

### Validation of the intact model

The mesh refinement was locally controlled at the highly stress-concentrated sites and the articulating surfaces. Using aspect ratio and Jacobian check, the quality of all elements were monitored to avoid sharp discontinuities and unrealistically high stress concentration. The mesh refinement was executed for modeling accuracy until excellent monotonic convergence behavior with < 1% difference in the total strain energy was achieved.

**Figure 3:**
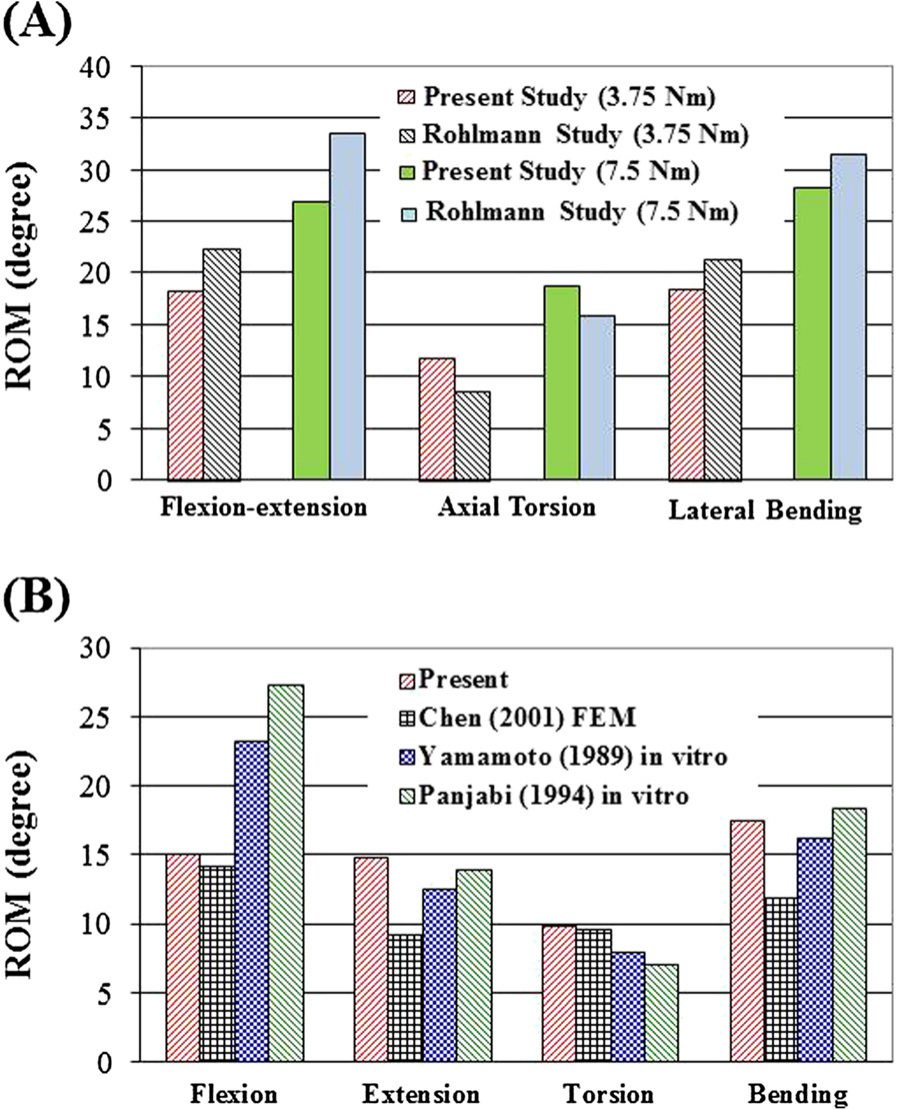
Comparison of ROM calculated for the five levels of intact lumbar spine (INT) with previous studies: (A) loading of 3.75 N-m and 7.5 N-m pure moments in the INT models; (B) loading of 10 N-m moments with 150 N preload in the INT models.

For model validation, ROM in five levels of the INT model was compared with Rohlmann’s *in vitro* study [29], which under different moments of 3.75 N-m and 7.5 N-m through the load control method. The data of present INT model were showed within the extreme values of the Rohlmann’s test (Figure 3A). However, under moment of 10 N-m with 150 N preload, the ROM is 6° to 11° lower than that from the *in vitro* test under flexion (Figure 3B). This might be explained by different preload application between the present (pressure preload) and earlier *in vitro* tests (vertical preload) [25,30,31]. The pressure preload calls for applying pressure at the top surface of L1 to create a compressive force of 150 N that is always perpendicular to the superior end of spinal column. Therefore, pressure preload causes much lower bending moments compared with the vertical preload. A higher bending moment might evaluate more ROM especially for long spine. In addition, facet contact force in torsion of each motion segment at the INT model is between 121 to 130 N and the values are demonstrated within the range of earlier studies [25,32] (Table 2). This INT model was therefore verified for further simulation analysis [26,28].

**Table 2:** **Comparison of facet contact forces under torsion between the present study and studies by Chen [**[18]**] and Shirari-Ad1 [**[32]**]. (unit: N)**

**Left torsion**	**Loading conditions**	**L1-2**	**L2-3**	**L3-4**	**L4-5**
Present study	10 N-m with 150 N preload	117	135	123	108
Chen’s	10 N-m with 150 N preload	121	157	161	155
Shirazi-Ad1’s study	10 N-m	107	123	117	78

### Boundary and loading conditions

Five muscle groups were iliopsoas (IP), multifidus (MF), longissimus (LG), iliocostalis (IC), and quadratus lumborum (QL) (Figure 1). Totally, there were forty-six muscles simulated in the finite-element analysis. A Cartesian coordinate system (*XYZ*) was established with the origin at the centroid of the L5 bottom to describe the muscle contractions (Figure 1). For the ligaments and muscles, the insertions and origins on the right and left sides were assumed symmetry with respect to the sagittal plane. For each muscle, the origin site was located onto the vertebral surface and the insertion site is cited from the literature studies [33,34].

The bottom surface of the S1 vertebral body was rigidly constrained for all models and the lumbar were flexed by the applied loads [28]. The loads include the weight compression and contractions of the abdominal muscles at the lumbar top and the muscular contractions along the lumbar column. In the INT and TLIF models, a 10-Nm moment were applied to mimic flexion, extension, left/right lateral bending, and left/right axial rotation, respectively. Bilateral bending and axial rotation were of concern due to asymmetrical positioning of implants in the current models. A 400-N compressive preload was used at the superior surface of L1 to mimic upper body weight. The TLIF cage models in conjunction with BPSF, UPSF, or UPSF plus a contralateral facet screw were loaded to determine their respective effects at the surgical level and in adjacent tissues. The criterion of controlling the same motion was adapted as a reasonable approach to evaluate the effects of implantation on the parafixed segments [28,35]. The iterative adjustment of the applied moment was used to control the same ROMs of the lumbar columns.

## Results

Analysis was conducted to show the difference in ROM, the maximum von Mises stresses of disc annulus, pedicle screw, and cage-vertebra interface on each TLIF construct. The data of INT model served as a baseline for interpretation of the results in the following sections. For example, ROM change rate = (ROM_TLIF_ - ROM_INT_) / (ROM_INT_) × 100 (%), where ROM_TLIF_ and ROM_INT_ represent the ROM for each motion segment in the instrumented TLIF models and in the INT model, respectively. First, this study presented the effects of the TLIF models under various cages positioning in conjunction with BPSF. Second, this showed the differences between UPSF and BPSF in the various TLIF cage models. Finally, this study evaluated the supplementary effect of a contralateral facet screw in the TLIFo model with UPSF.

### Effects of different cage positioning in the TLIF models with BPSF

**Figure 4:**
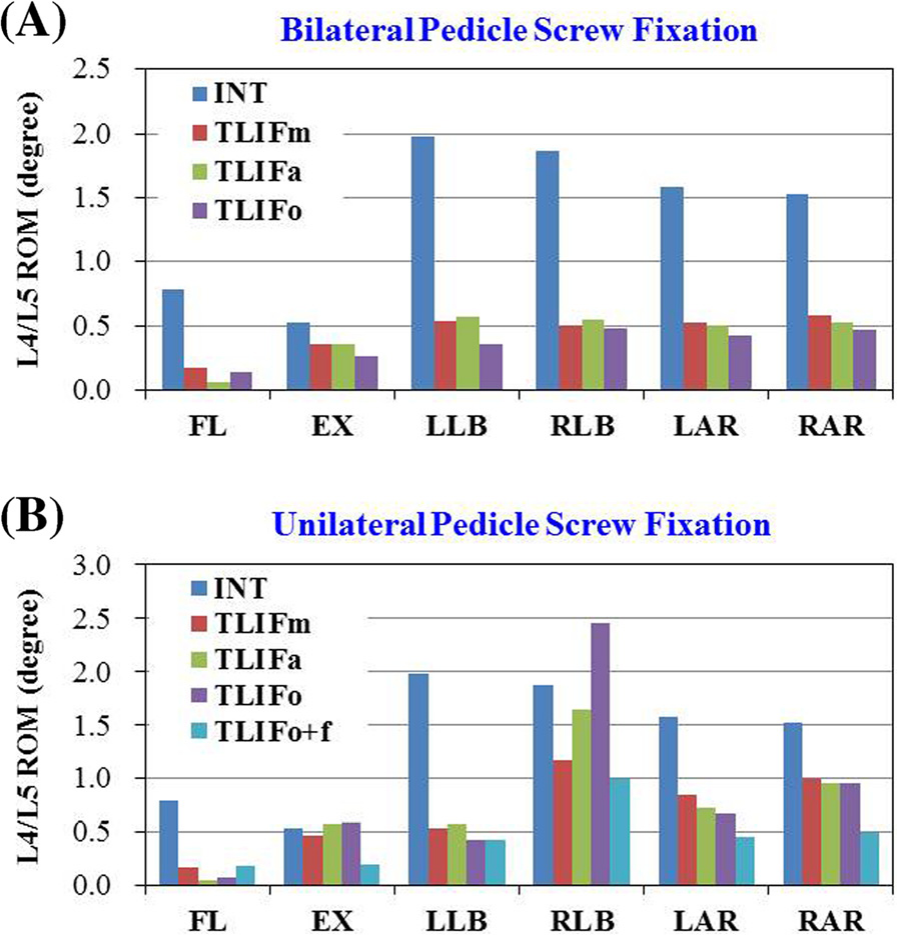
** The calculated ROM at surgical L4-5 level in the INT, TLIFm, TLIFa, TLIFo models supplemented with: (A) Bilateral pedicle screw fixation or (B) Unilateral pedicle screw fixation.** Translaminar facet screw was used in the model of TLIFo plus unilateral pedicle screw fixation (TLIFo + f). (Fl = Flexion; Ex = Extension; LLB = Left lateral bending; RLB = Right lateral bending; LAR = Left axial rotation; RAR = Right axial rotation).

**Figure 5:**
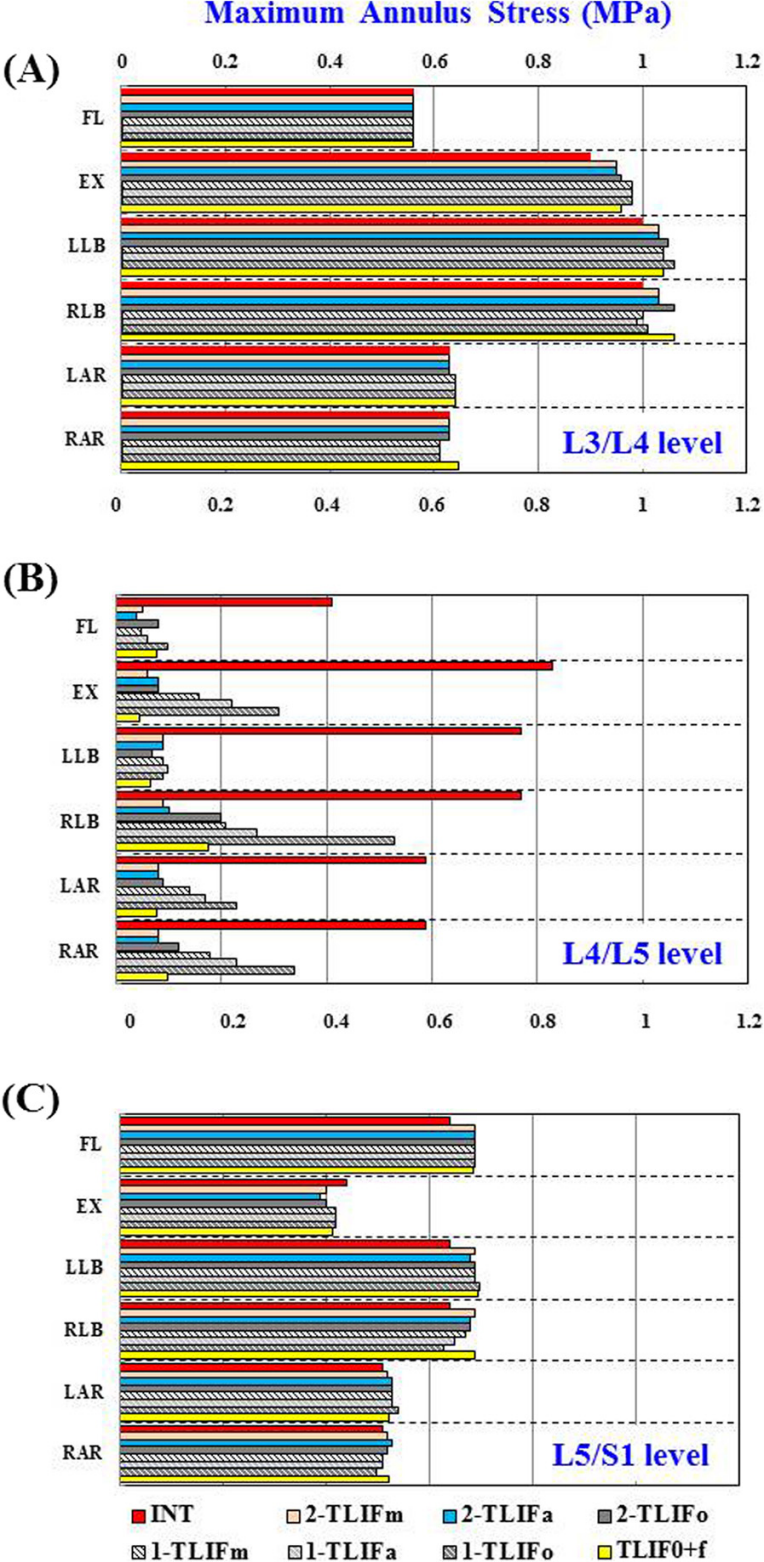
The maximum von Mises stress of annulus in all models (1 = with unilateral pedicle screws fixation; 2 = with bilateral pedicle screw fixation).

Among all the TLIF models with BPSF, similar ROM (<5%) in all motions were observed at surgical levels, except TLIFa in flexion (61% lower) and TLIFo in left lateral bending (33% lower), as compared with the TLIFm model (Figure 4A). Similar annulus stresses at surgical level were observed in all the TLIFa and TLIFm models; however, the TLIFo model had 8% higher stress in flexion, 6% higher in right axial rotation, and 14% higher in right lateral bending at surgical level, as compared with the TLIFm model (Figure 5B). By using BPSF, ROM and annulus stresses at adjacent levels did not show obvious differences among these models (Figures 5A and 5C). Asymmetrical cage positioning in the TLIFo model with BPSF induced slightly uneven motion and annulus stresses at surgical level in flexion, contralateral axial rotation and lateral bending.

### Differences of UPSF versus BPSF in the TLIF models

All the TLIF models supplemented with left UPSF increased motion in extension (15 ~ 22% higher), right axial rotation (26 ~ 32% higher), and right lateral bending (27 ~ 59% higher) at surgical level, as compared with each TLIF model plus BPSF (Figures 4A-B ). In particular, the TLIFo model with UPSF increased motion at surgical level mostly in right lateral bending (59% higher) and right axial rotation (32% higher).

**Figure 6:**
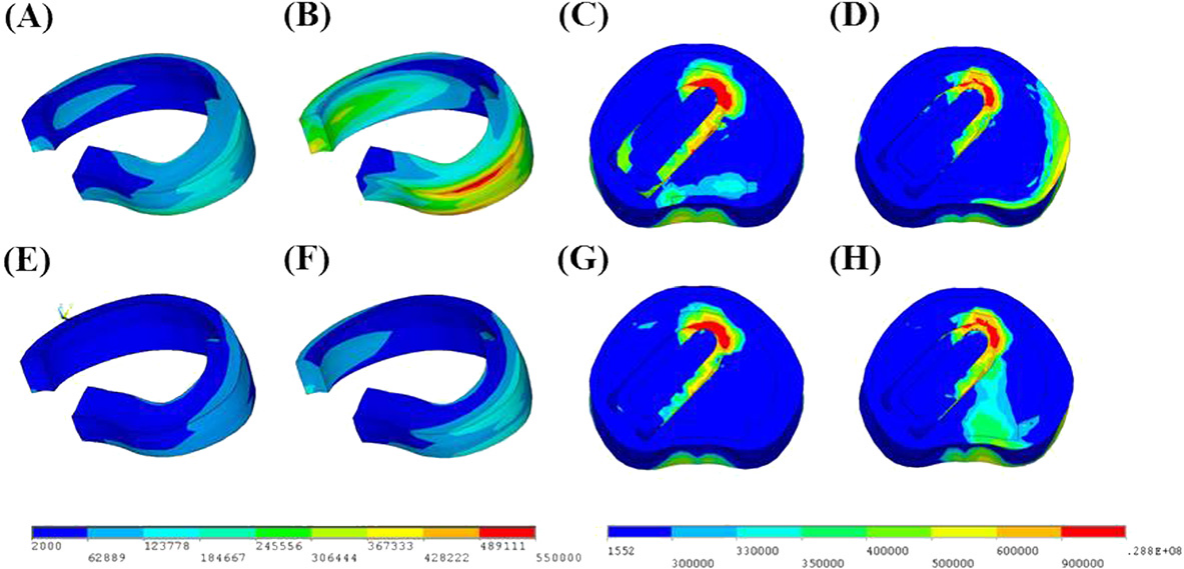
** For the unilateral fixation, the stresses distribution in annulus at surgical level of TLIFo model under (A) right axial rotation (RAR) or (B) right lateral bending (RLB), and the cage-bone interface under (C) RAR or (D) RLB.** The stresses distribution in annulus at surgical level of TLIFo model supplemented with a translaminar facet screw contralaterally under **(E)** RAR or **(F)** RLB, and the cage-bone interface under **(G)** RAR or **(H)** RLB.

All the TLIF models supplemented with left UPSF increased annulus stresses in extension (12 ~ 33% higher), right lateral bending (14 ~ 45% higher), left axial rotation (11 ~ 27% higher), and right axial rotation (14 ~ 38% higher) at surgical level, as compared with each TLIF model plus BPSF (Figure 5A). In particular, the TLIFo model with UPSF had the greatest variability of annulus stresses at surgical level during extension (29% higher), right lateral bending (41% higher), left axial rotation (25% higher), and right axial rotation (39% higher). As shown in Figures 6A-D, the prominent stress was demonstrated at the outside edge of L4/L5 annulus, near both endplates of cage-vertebra interface in contralateral axial rotation and lateral bending.

**Figure 7:**
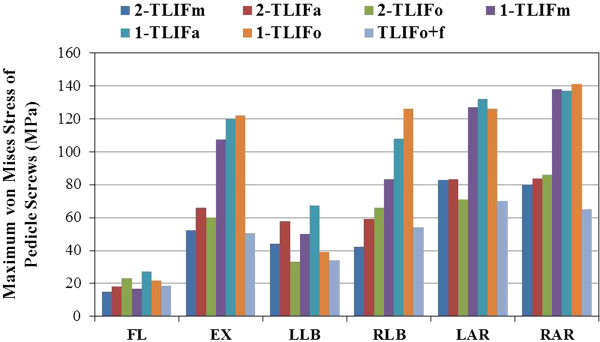
** The maximum von Mises stress of pedicle screws in all models.** (1 = with unilateral pedicle screws fixation; 2 = with bilateral pedicle screw fixation) (FL = Flexion; EX = Extension; LLB = Left lateral bending; RLB = Right lateral bending; LAR = Left axial rotation; RAR = Right axial rotation).

In addition, the stresses of pedicle screws were analyzed to reveal the differences among the TLIF cage models in conjunction with UPSF *versus* BPSF (Figure 7). Different cage positioning altered screw stress; however, UPSF used in TLIF cage models increased screw-stress much more than BPSF, especially in extension (69 ~ 85% higher), right lateral bending (79 ~ 85% higher), and right axial rotation (54 ~ 77% higher), as compared with each TLIF model plus BPSF. In particular, the TLIFo model with UPSF had the highest screw-stresses in extension (85% higher), right lateral bending (85% higher), and right axial rotation (77% higher). Asymmetrical positioning of TLIFo with UPSF is not favored due to prominent increase in ROM, and stresses shielded to the contralateral annulus, cage-vertebra interface, and pedicle screw at surgical level.

### Effects of contralateral facet screw supplemented in the TLIFo model with UPSF

Supplementary facet screw fixation on the contralateral side was done to evaluate the biomechanical behavior after TLIFo plus UPSF procedure. In this TLIFo + f model, ROM at surgical level decreased by 5% in flexion, 57.0% in extension, 26% in left lateral bending, 15% in right lateral bending, 46% in left axial rotation, and 51% in right axial rotation, as compared with the TLIFo model (Figure 4B).

The maximum annulus stresses at surgical level of the TLIFo + f model decreased by 82% in flexion, 93% in extension, 92% in left lateral bending, 78% in right lateral bending, 82% in left axial rotation and 86% in right axial rotation, respectively, as compared with the INT model (Figure 5A). As shown in Figures 6E-H of the TLIFo + f model, a supplementary facet screw decreased annulus stresses in contralateral axial rotation and lateral bending, and the stress of the cage-vertebra interface was shifted medially and posteriorly. In addition, by using a facet screw in TLIFo + f model, ROM and annulus stress at surgical or adjacent levels did not show obvious difference, as compared with the TLIF models plus BPSF (Figures 5B-C).

## Discussion

Interbody cages for spinal fusion have been a promising innovation; nevertheless, there is ongoing debate regarding the necessary conditions for successful arthrodesis and adjacent level effect. The influences of surgical approach, additional posterior instrumentation, implant design, and bone mineral density on the stiffness, compressive strength and three-dimensional flexibility of the spinal units have been demonstrated under static and cyclic loading [3-5,7,8,10,19,20,22,23,28,36-38]. The geometry of TLIF cages, including shape, length, width, and serrated surface profile, does not affect construct behavior when the cages are used in conjunction with posterior fixation [7,10,12,32,39]. Minimal cage migration under cyclic loading was attributed to the compression force applied during pedicle screw tightening. The risk of cage subsidence is higher, especially for the patients with poor bone quality [3,5,19,27,38]. Therefore, the operated segment should provide enough cage-endplate contact stresses under compression to prevent TLIF cage displacement and allow spaced graft consolidation, while maintaining structural flexibility that approaches the intact specimen to decrease adjacent segment degeneration [22,24,32,40,41]. The present simulation study used the motion-controlled method to investigate the performance of TLIF cages implanted in different positioning and supplemented with BPSF, UPSF or UPSF plus facet screw. Among all the TLIF cage models with BPSF or UPSF, a diagonal cage had less ROM control in flexion, contralateral axial rotation and lateral bending than the other cages positioned. When the TLIFo model was used with left UPSF, the lowest ROM control at surgical level was found in right lateral bending. Supplementation of a contralateral facet screw in the TLIFo model with UPSF shared stresses around the neighboring tissues and revealed similar stress pattern to those models with BPSF.

Harris *et al*. used the T12-S1 cadaveric spine to reveal that axial rotation increased at the segment of a TLIF stand-alone cage, UPSF further increased stiffness, and BPSF approximated axial rotation most closely to the intact specimen [40]. Chiang *et al*. used a 3-level FE model and found that a single oblique cage from the posterior approach (- like a TLIFo procedure) combined with BPSF gained approximate biomechanical behavior, but slightly greater subsidence and increased screw stress than the two parallel cages model [27]. This study demonstrated that symmetrical positioning of the TLIF cages and BPSF contributed similar annulus stress at the surgical or adjacent segment; while asymmetrical positioning of a diagonal cage plus UPSF in the TLIF model showed ROM increased and stresses concentrated on the neighboring pedicle screw, annulus and cage-endplate interface mostly in contralateral axial rotation and lateral bending. This procedure might induce screw breakage, contralateral radiculopathy, and cage micromotion, as in the reported complications of minimal access TLIF technique using UPSF [11,12,14-16,18,38]. Relative micromotion and stress concentration on the cage-vertebral junction possibly hinder bone growth into the surface pores of interbody spacers and eventually induce cage migration with the endplate failure [3,5,17,37,38]. However, an FE analysis study cannot ascertain the level of stiffness required to obtain long-term stability for solid fusion.

Bilateral pedicle screw constructs are the standard for instrumentation, providing rigid fixation and increased fusion rates [7,8]. Suk *et al*. reported comparable clinical outcomes and fusion rates using UPSF versus BPSF in patients undergoing one- and two-level posterolateral lumbar fusions [41]. In the evolution of PLIF or TLIF construct for load sharing and disc height restoration, the role of posterior instrumentation has changed from principal load-bearing to that of tension band and neutralization. Goel demonstrated that unilateral constructs were subject to coupled motions as a result of the inherent asymmetry, which unlikely provided enough rigidity for decompressive procedures that required a complete excision of the disc [36,39]. Slucky *et al*. found that unilateral pedicle screw constructs after the TLIF provided only half of the improvement in stiffness compared to bilateral constructs, and a significant off-axis rotational motion could be detrimental to stability and fusion [17]. With the advent of minimally invasive TLIF techniques to achieve spinal fusion, it become essential to have lesser soft tissue dissection, low implant load without compromising the spinal stiffness, and reduction of surgical time, postoperative pain and hospitalization. The advantage of a unilateral pedicle screw and contralateral facet screw construct allows 270° interbody and posterolateral fusion with little muscle stripping. In an event of increasing compressive load and posterior implant used, improvements in the cage-endplate contact stresses and the underlying bone quality could overcome excessive cage micromotion, leading to the TLIF constructs stabilized [3,7,25,38]. However, age-related changes in the mechanical properties of annular fibers and vertebral bone reduce the stability of TLIF cage on a spinal segment and increase early failure of the endplate [3,5,7,21,24,29,41,42]. Therefore, the minimally invasive TLIF surgery is not favored for implanting a diagonal cage and UPSF in the osteoporotic spine, and contralateral facet screw fixation is suggested to provide the same effectiveness as the conventional methods used [17,21,42].

Several limitations in this study are related to the slightly simplified and idealized material properties of simulation, such as the nonlinear behavior of spinal ligaments, the viscoelasticity of intervertebral disc, and the grades of degeneration - these all differ from cadaver specimens [24,25,42]. Degenerative disc is common in most patients before surgery; however, it is challenging in modeling to assign material properties to various grades of degenerative disc, such as delamination, dehydration or reduced disc height. Therefore, FE models should be interpreted as a trend only because of the variability of different human tissues. Also, the current authors have not mentioned the bone growth into the cage, and the ligament pretension after inserting implants. The loading conditions in the present simulation were similar to those of the traditional *in vitro* tests; thus, this study did not consider muscle contraction and complicated external load conditions [29].

## Conclusions

In conclusion, TLIF surgery is not favored for implanting a diagonal cage and UPSF, as ROM increased and stresses concentrated on the neighboring annulus, cage-vertebral interface, and pedicle screw in contralateral axial rotation and lateral bending. Supplementation of a contralateral facet screw is recommended for the use of this construct.

## Competing interests

All authors do not have the financial and personal relationships with the organization that could inappropriately influence (bias) the current research. No disclosure of commercial interests and no conflicts of interest in this study.

## Authors’ contributions

SHC, SCL, and CWW conceived of the study, participated in the design of the study and performed the data analyses. SHL, CWW, and SHC developed the model and drafted the manuscript with the help of SHC and WCT. All authors carried out the analyses, read, and approved the final manuscript.

## Pre-publication history

The pre-publication history for this paper can be accessed here:

http://www.biomedcentral.com/1471-2474/13/72/prepub
